# Fabrication of Hybrid Materials for Catalysis

**DOI:** 10.3390/molecules31081295

**Published:** 2026-04-16

**Authors:** Jerry J. Wu, Michael Arkas, Dimitrios A. Giannakoudakis

**Affiliations:** 1 Department of Environmental Engineering and Science, Feng Chia University, Taichung 40724, Taiwan; jjwu@fcu.edu.tw; 2 Institute of Nanoscience and Nanotechnology, National Centre of Scientific Research “Demokritos”, 15310 Athens, Greece; 3 School of Chemistry, Aristotle University of Thessaloniki, 54124 Thessaloniki, Greece; dgiannakoudakis@ichf.edu.pl

## 1. Introduction and Scope

The catalytic enhancement of chemical reactions is crucial for promoting environmental remediation in soil, air, and water, as well as for clean energy production and overall sustainable development. Designing innovative, stable, selective, and reusable catalysts with higher activity remains a critical goal of current research. The current trends in formulating and synthesizing composite catalytic systems that encompass a variety of properties and multiple functionalities are summarized in this Special Issue, entitled “Fabrication of Hybrid Materials for Catalysis.” Nanostructured hybrid organic and inorganic materials offer unlimited potential to fine-tune catalytic conduct at the molecular and atomic scales. Novelties in assembly methods—for instance, hydrothermal [1], ultrasound [2], and microwave-assisted synthesis [3]; coprecipitation [4]; self-assembly [5]; inorganic shell formation onto organic scaffolds [6]; and heterojunctions [7]—allow the development of catalysts with accurately engineered structures and precisely distributed active sites. Furthermore, they tend to employ fewer toxic reactants and solvents and ambient temperatures, rendering the overall processes eco-friendly and sustainable. These research breakthroughs are transforming conventional catalytic setups into faster, more efficient, recyclable, and environmentally friendly systems. This collection features selected research articles and communications on new synthetic paths, characterization techniques, and applications. Some of the topics covered include doped ceramics, polyoxometalates, layered double hydroxides, metal–organic frameworks, bimetallic composites, core–shell nanocomposites, metal alloys dispersed into active carbon, graphene oxide decorated with quantum dots, and multifunctional hybrid systems. Applications discussed include clean energy production [8,9], pollutant neutralization [10], and chemical synthesis [11]. Along with our acknowledgments to all of the authors and reviewers for their invaluable cooperation, we hope that this collection will spark interest in extensive interdisciplinary research, thereby accelerating the implementation of real-world necessities in this field.

## 2. Overview of the Published Articles in the Current Research Framework

In assessing the feasibility of replacing fossil fuels with hydrogen, much recent research focuses on identifying suitable electrocatalysts for the electrolytic decomposition of water. Layered double hydroxides (LDHs) present a cost-effective solution. NiFe-based LDHs exhibit improved oxygen evolution reaction kinetics, as described in [Contribution 1] [12]. This is achieved by incorporating Mo, which enhances the active surface of NiFeMo-LDH nanosheet arrays and increases the electrocatalyst’s electron transport efficiency and overall reactivity. The same principle may be applied to another intriguing development, i.e., the production of bifunctional or multifunctional catalysts that may mediate completely different reactions [13]. As a plausible implementation, [Contribution 2] proposes a bifunctional NiCr LDH formed by the hydrothermal coprecipitation of nickel and chromium nitrates [14], which is further decorated by sol-immobilization with palladium and silver nanoparticles, yielding Ag@NiCr LDH and Pd@NiCr LDH composites. These are tested in the oxidation of CO and the reduction of nitrobenzene to aniline by NaBH_4_. The formation of Cr^3+^/Cr^6+^ @Pd° ion pairs that function as charge transfer centers dramatically enhances the redox activity of Pd@NiCr LDH. A similar hydrothermal method is employed for the preparation of metal–organic frameworks (MOFs) [15] such as the core–TiO_2_ shell MIL-101(Cr)@TiO_2_ multifunctional composite disclosed in [Contribution 3] and tested to determine the photocatalytic degradation of dyes (Rhodamine B, methylene blue) and exhaust (NO_x_, CO, and C_3_H_6_) pollutants. The MOF matrix efficiently facilitates the dispersion of TiO_2_ nanoparticles by impeding their agglomeration while broadening their effective absorption range to visible light, introducing potential treatments for industrial wastewater and vehicle exhausts.

In general, composites based on metal and metalloid oxide matrices constitute a class of multifunctional catalysts, with numerous recent applications in oxidative, reductive, and photocatalytic processes. Two nickel catalysts, supported on alumina trilobe and alumina tablet, are employed for in situ catalyst characterization as reported in [Contribution 4], accomplished using pulse chemisorption, temperature-programmed oxidation and reduction, and CO_2_ hydrogenation [16]. In addition to the slow deposition of graphite, the deactivation of the spent catalysts is attributed to their reduced dispersion. Some unconventional production methods of ceria-doped SiO_2_ are included in [Contribution 5], wherein ultrasound micromixing and microwave radiation dielectric heating [17] using an open-end coaxial antenna are introduced into the production of classic solvothermal mesoporous materials. The structure of the support is directed by micellar formation, resulting in mesoporous silica rich in homogenously dispersed cerium. The sonochemically produced sieves are used as support for a nickel catalyst for the ethanol steam reforming reaction that achieves complete ethanol conversion and high H_2_ selectivity (65%). By achieving precise control over the architecture and the active sites of this composite, this method gives insights into controlled synthesis and modular design, one of the prominent areas of modern catalysis. In an analogous framework, the functionalization of a cheap adsorbent with a common photocatalyst leads to a vermiculite/TiO_2_ composite according to [Contribution 6]. The hybrid is used to eliminate methylene blue by chemisorption and photodegradation. At the optimal clay/ceramic ratio of 1:5, synergetic effects such as the homogenous dispersion of TiO_2_ to the layered structure of vermiculite raise decomposition to 91.24%, which is a 5.3-fold increase in comparison to pure TiO_2_ due to optical band gap narrowing and enhanced absorption in the visible light. For the photocatalytic disintegration of a second model dye, i.e., methyl orange dye, with simultaneous hydrogen production [18], a hybrid microstructure catalyst is formed employing the photosensitizer meso-tetra(p-hydroxyphenyl) porphine as a matrix for TiO_2_ growth in the form of anatase. The combination with a Pt co-catalyst creates a series of beneficial effects [Contribution 7]: (a) the extension of the titania absorption to the visible light, (b) the effective splitting of the electrons and holes of the organic scaffold, and (c) photoinduced electron transport to the surface of platinum for efficient water splitting. The doping of photocatalytic metal oxide is a common strategy to enhance its performance. Specifically, decorating ZnO_2_ with Ba^2+^ cations, as presented in [Contribution 8], increases hydrophilicity and surface area and enhances optoelectronic properties, and thus photocatalytic activity [19]. Moreover, by creating protection from the harsh catalytic conditions and enhancing long-term stability, this work contributes to our understanding of photocorrosion procedures. Another example of visible light-photoinduced pollution remediation involves plasmonic photocatalysis mediated by Au nanoparticles dispersed into ZnO nanosheets [20]. In a mechanistic study [Contribution 9], the decomposition of methyl orange and Rhodamine B is investigated both theoretically and experimentally as a function of metal nanoparticle distribution, irradiation wavelength, and electric field intensity.

Of particular interest are inorganic matrices based on carbon allotropes, heterogeneous compounds with similar structures, and their derivatives. [Contribution 10] proposes impregnation calcination to incorporate alloys of Fe, Cu, Mn, and Ce into activated carbon. The derived Fenton catalysts, designed to neutralize organic pollutants in wastewater by generating free radicals from H_2_O_2_ [21], prove to be an effective option, eliminating more than 90% of methylene blue even after 10 catalytic cycles. In a second instance, a composite electrochemical sensor is fabricated by modifying a glassy carbon electrode with multi-walled carbon nanotubes bearing graphene oxide quantum dots. The result in [Contribution 11] is applied for the detection of adenine, thymine, guanine, and cytosine in artificial saliva samples by oxidation employing differential pulse voltammetry [22]. In a dual-photocatalytic implementation that addresses both energy production via hydrogen generation and environmental remediation via Rhodamine B degradation, a three-dimensional carbon nitride network is synthesized via thermal polymerization, as discussed in [Contribution 12]. The interconnected porous architecture yields a large specific surface area with abundant active sites that facilitate the separation of photogenerated charge carriers.

[Contribution 13] attempts to address a gap in our understanding of how interfaces in composite catalysts influence charge transfer. In a typical water disinfection application [23] using solar energy, Bi_2_MoO_6_ nanosheet evolution onto Bi_5_O_7_I microrods results in a Bi_2_MoO_6_/Bi_5_O_7_I Z-scheme heterojunction. The composite is employed for the inactivation of *E. coli* through h^+^, •O_2_^−^, and •OH production that oxidizes the bacterial membrane and causes apoptosis. The Kraft oxidative depolymerization of phenethoxybenzene by a Keggin-type polyoxometalate (TBA)_5_[PMo_10_V_2_O_40_] [24] is monitored as a case study for lignins bearing β-O-4 bonds.

[Contribution 14] determines the optimal temperature, reaction time, and catalyst amount with response surface methodology, and the most favorable combination yields 75.8% conversion. Finally, [Contribution 15] discusses the assembly of heterostructures, i.e., different intercalated crystallization motifs exhibiting outspread interfaces and elegant architectures, which is an engaging line of action for accelerating photocatalyst performance [25]. A composite constructed by BiOCl nanoflakes and FeOCl nanospindles is tested for the photocatalytic elimination of Rhodamine B, and the optimal formulation containing 15% BiOCl increases the activity of pure FeOCl 90-fold by enabling the separation of photoinduced carriers.

## 3. Conclusions and Perspectives

Hybrid and composite materials represent a distinct category of catalysts, offering alternative directions for producing clean energy, mitigating environmental pollution, and performing thermodynamically unfavorable chemical synthesis. Despite the promising recent advances, deeper knowledge of critical issues is required to unlock their full potential. Future research should focus on gaining a deeper understanding of the various mechanisms involved, optimizing theoretical predictions, and engineering higher stability. This will be accomplished by developing novel characterization and monitoring techniques, design strategies based on molecular simulations, and artificial intelligence. Sustainability is also a major factor, postulating the replacement of scarce materials and avoiding toxic reactants or byproducts and energy-consuming methods. The combination of these efforts with the development of upscaling protocols will open up new horizons for actual industrial applications.
